# Hospital cost structures and resource utilisation across representative treatment pathways relevant to complex orthopaedic infection care: a nationwide DRG-based analysis in Germany

**DOI:** 10.1007/s00068-026-03391-2

**Published:** 2026-11-10

**Authors:** Houmam Anees, Goran Georgievski, Jonas Pawelke, Matthias Muelke, Gero Knapp, Thaqif El Khassawna, Markus Rupp, Christian Heiss

**Affiliations:** 1 Department of Trauma, Hand and Reconstructive Surgery, Faculty of Medicine, Justus-Liebig-University of Giessen, 35392 Giessen, Germany; 2 Experimental Trauma Surgery, Faculty of Medicine, Justus-Liebig-University of Giessen, 35392 Giessen, Germany; 3 School of Pharmacy, The University of Jordan, Amman, 11942 Jordan; 4 Department of Orthopaedics and Traumatology, Faculty of Medicine, Biruni University, Istanbul, Türkiye

**Keywords:** Implant-associated infection, Periprosthetic joint infection, Fracture-related infection, Hospital costs, Diagnosis-related groups, Health economics

## Abstract

**Purpose:**

Implant-associated orthopaedic infections require prolonged inpatient treatment, repeated surgery and multidisciplinary care, yet nationwide evidence on how hospital resources are distributed across clinically distinct treatment pathways is limited. This study characterised hospital-side case costs, resource utilisation and aggregate expenditure across representative German diagnosis-related groups (DRGs) relevant to complex orthopaedic infection care.

**Methods:**

Publicly available 2024 data from the German Institute for the Hospital Remuneration System (InEK) were analysed. Twelve representative DRGs were selected a priori from official definitions and diagnostic/procedural profiles and grouped into six treatment pathways. The InEK cost matrix reflects standardised hospital-side resource costs from participating calculation hospitals and does not represent actual DRG reimbursement. National case frequencies, length of stay and mean case costs were analysed using frequency-weighted pathway estimates, while pathway-level cost composition was calculated from aggregate component expenditure.

**Results:**

The included DRGs represented 36,212 hospitalisations and €305.2 million in aggregate annual hospital-side expenditure represented by the selected DRGs. Mean hospital-side costs ranged from €5,859 to €22,495 per case. Ward care accounted for approximately 34–69% of pathway expenditure; operating theatre services contributed up to 39% in revision arthroplasty. Geriatric rehabilitation generated the largest aggregate expenditure (€119.0 million; 39.0%) because of high case volume, whereas complex vacuum-assisted wound care had the highest per-case cost but only 2.9% of aggregate expenditure. At the aggregated DRG level, mean length of stay was associated with mean case cost (Pearson *r* = 0.87, 95% CI 0.60–0.96; *P* < 0.001); leave-one-out correlations ranged from *r* = 0.79 to *r* = 0.96.

**Conclusion:**

Resource requirements differ markedly across representative DRG pathways relevant to complex orthopaedic infection care. Because the selected DRGs are not infection-exclusive, the reported expenditure should not be interpreted as the national economic burden of implant-associated orthopaedic infection. The findings may inform hospital resource planning and future cost–revenue analyses.

**Supplementary Information:**

The online version contains supplementary material available at https://doi.org/10.1007/s00068-026-03391-2.

## Introduction

Diagnosis-related group (DRG)-based payment systems require transparent information on the resources consumed by hospital care. In Germany, the G-DRG cost-accounting framework allocates DRG-relevant hospital costs to standardised cost centres and cost categories and uses these data to derive relative cost weights for inpatient remuneration [1, 2]. Importantly, the cost data used for calculation and the reimbursement ultimately received by a hospital are related but not identical.

Implant-associated orthopaedic infections, including periprosthetic joint infection (PJI), fracture-related infection (FRI), osteomyelitis and infection-associated soft-tissue defects, are particularly resource intensive. Contemporary studies consistently report prolonged hospitalisation, repeated operations and substantially higher costs than uncomplicated musculoskeletal care [3, 4]. A recent European systematic review found substantial direct hospital costs for hip and knee PJI, with pronounced variation according to debridement, antibiotics and implant retention (DAIR), one-stage exchange and two-stage exchange strategies [5].

The scale of this burden is amplified by treatment volume. In the United States, the economic burden of PJI has risen over time and is projected to increase further, largely because of the growing number of arthroplasties and infection episodes [6, 7]. Across Europe, modelling based on procedure volumes and national reimbursement data estimated a PJI reimbursement burden exceeding €346 million for primary hip and knee arthroplasties performed in 2019 [8]. In trauma surgery, German and international studies similarly demonstrate that FRI substantially increases hospital resource use and treatment costs [3, 9].

Our previous nationwide study in Germany focused on the epidemiology of implant-associated complications from 2000 to 2023 and used three illustrative DRGs to demonstrate broad differences in contemporary resource utilisation [10]. It did not, however, examine detailed cost-centre composition, treatment-pathway-specific cost structures or the interaction between per-case cost intensity and national treatment volume.

The present study therefore aimed to provide a dedicated nationwide DRG-based analysis of representative pathways relevant to complex orthopaedic infection management. Specifically, we sought to quantify hospital-side case costs, characterise the distribution of expenditure across hospital cost centres and cost categories, compare weighted resource profiles across clinically defined pathways, assess the association between length of stay and case costs, and estimate aggregate annual expenditure represented by the included DRGs.

## Methods

### Study design and data sources

This cross-sectional health-economic analysis used publicly available administrative data from the German Institute for the Hospital Remuneration System (Institut für das Entgeltsystem im Krankenhaus; InEK) for the 2024 aG-DRG system [11]. National DRG frequencies and diagnostic/procedural characteristics were obtained from the InEK Data Browser, and DRG-level mean hospital-side case costs and component costs were taken directly from the corresponding InEK cost matrix. No cost estimates were derived from DRG relative weights or base rates.

The analysis was conducted from the hospital perspective. The reported cost values reflect DRG-relevant hospital-side case costs submitted by participating calculation hospitals to InEK under a standardised full-cost accounting framework. These data form the empirical basis for deriving DRG relative cost weights but do not represent the actual DRG reimbursement paid by statutory health insurers. Actual case-based remuneration depends on the applicable relative weight and base rate and may additionally be affected by supplements and other payment components [1, 2]. This distinction was maintained throughout the analysis. Accordingly, the reported values represent standardised DRG-relevant inpatient resource costs rather than hospital revenue or payer expenditure. Costs outside the represented inpatient DRG episode, including outpatient care, post-discharge expenditure, societal costs and productivity losses, were not captured.

### Selection and classification of representative treatment pathways

Representative DRGs were selected a priori on the basis of their official 2024 G-DRG definitions and the diagnostic and procedural spectrum reported in the InEK Data Browser. Eligible DRGs had to represent at least one resource-intensive component commonly encountered in complex orthopaedic infection management: revision arthroplasty, septic hip/femur procedures, bone and joint infection, soft-tissue reconstruction, complex vacuum-assisted wound care or geriatric early rehabilitation. Selection was intended to capture clinically relevant resource-utilisation pathways rather than to create an exhaustive or infection-exclusive case definition. Selection was based on the presence of infection-relevant diagnoses or procedures where available, or on an explicit role of the DRG in resource-intensive treatment components frequently required during complex orthopaedic infection care.

Twelve DRGs were included and assigned to six predefined pathways (Table 1). Infection relevance was supported by the official DRG descriptors and by the corresponding diagnostic/procedural profiles. For each included DRG, Supplementary Table S1 provides the official 2024 G-DRG definition, relevant diagnostic or procedural features identified in the InEK Data Browser where available, the assigned treatment pathway, and the specific rationale for inclusion. For example, T84.5 (infection and inflammatory reaction due to internal joint prosthesis) was among the most frequent secondary diagnoses in the revision arthroplasty groups I01Z, I03B and I04Z, occurring in 70.8%, 35.8% and 47.4% of cases, respectively. Several DRG definitions also explicitly incorporated bone/joint infection, spacer procedures, osteomyelitis, complex diagnoses or prolonged vacuum-assisted treatment, supporting their inclusion as clinically relevant components of complex infection management. Nevertheless, the selected groups were not restricted to confirmed implant-associated infections, a limitation considered in the interpretation of aggregate expenditure.

**Table 1:** Characteristics of the twelve representative diagnosis-related groups

DRG	Short clinical descriptor	Cases	Mean LOS (days)	Mean hospital-side cost (€)
I98Z	Complex vacuum-assisted wound treatment	389	35.3	22,495
I01Z	Complex major lower-extremity joint procedures	620	28.4	19,147
I12A	Bone/joint infection; extremely severe CC	347	24.6	16,972
I22B	Tissue/skin transplantation; complex reconstruction	969	23.0	14,072
I08C	Complex hip/femur procedures including spacer procedures	991	17.8	13,524
I04Z	Knee endoprosthesis implantation/exchange/removal with complicating diagnosis	2,067	15.1	12,891
I08D	Hip/femur procedures with complex diagnosis/procedure	4,118	15.5	11,714
I03B	Hip revision/replacement with complex diagnosis/procedure	1,267	16.1	11,675
I50A	Tissue/skin transplantation or prolonged VAC treatment	1,498	17.9	11,202
I12B	Bone/joint infection with severe CC; revision/spacer/osteomyelitis features	970	16.6	10,374
I12C	Bone/joint infection with severe CC; without revision/spacer/osteomyelitis	2,658	10.4	6,060
I41Z	Geriatric early rehabilitation for musculoskeletal disorders	20,318	18.8	5,859

Descriptors are shortened English summaries of the official 2024 G-DRG definitions. Treatment-pathway assignment is shown in Table 2. Full official DRG definitions, relevant diagnostic/procedural features and the rationale for inclusion are provided in Supplementary Table S1. CC, complications or comorbidities; LOS, length of stay; VAC, vacuum-assisted wound care

### Hospital cost and resource-utilisation analysis

Hospital-side case costs were evaluated at two complementary levels. First, costs were grouped by standardised hospital cost centre, including ward care, intensive care, operating theatre, anaesthesia, radiology, laboratory, therapeutic services and other hospital services. Second, costs were evaluated by standardised cost category, including personnel, pharmaceuticals, implants and other medical materials, as well as medical and non-medical infrastructure. For each DRG, absolute component costs were obtained directly from the InEK cost matrix and the proportional contribution of individual components was calculated relative to total DRG-relevant case costs. Complete DRG-level cost-centre and cost-category components are provided in Supplementary Tables S3 and S4, respectively.

DRG-level values were subsequently aggregated within each clinical pathway. Weighted mean case cost and weighted mean length of stay were calculated using national DRG frequencies as weights. For pathway-level cost-centre and cost-category composition, absolute component expenditure was calculated for each DRG by multiplying the mean component cost by the national number of hospitalisations. Pathway-level component shares were then calculated as the summed component expenditure across DRGs divided by the summed total hospital-side expenditure of the corresponding pathway. Thus, component shares were weighted by both case volume and DRG-specific cost rather than by case frequency alone. Aggregate annual expenditure represented by the included DRGs was estimated as the national number of hospitalisations multiplied by the corresponding mean hospital-side case cost. The expenditure share of each pathway was calculated relative to the total expenditure represented by all twelve included DRGs. These estimates should not be interpreted as the total national cost of implant-associated orthopaedic infection, because the selected DRGs are representative rather than infection-exclusive.

### Outcomes and statistical analysis

The primary outcome was mean hospital-side cost per hospitalisation. Secondary outcomes were weighted pathway-level cost and length of stay, cost-centre and cost-category composition, aggregate annual expenditure represented by the included DRGs, and pathway expenditure shares.

Descriptive statistics were used to summarise costs and resource utilisation. The association between mean length of stay and mean case cost across the twelve DRGs was assessed using Pearson’s correlation coefficient. Spearman’s rank correlation coefficient was calculated as a sensitivity analysis. To assess the influence of individual DRGs, a leave-one-out sensitivity analysis was performed by repeating the Pearson correlation after sequential exclusion of each DRG. Because geriatric rehabilitation (I41Z) was the highest-volume pathway and was not infection-specific, a sensitivity analysis excluded I41Z and recalculated the aggregate expenditure and expenditure shares of the remaining five pathways (Supplementary Table S2). Correlation analyses were performed at the aggregated DRG level rather than at patient level. All tests were two-sided and *P* < 0.05 was considered statistically significant. Analyses and visualisations were performed in R version 4.6.1 (R Foundation for Statistical Computing, Vienna, Austria).

### Ethical considerations

Only anonymised, aggregated and publicly available administrative data were analysed. No individual-level patient information was accessed. Ethical approval and informed consent were therefore not required.

## Results

### Characteristics of included DRGs and treatment pathways

The twelve included DRGs represented 36,212 hospitalisations. Across all groups, aggregate annual hospital-side expenditure represented by the selected DRGs was €305.2 million, the frequency-weighted mean cost was €8,428 per hospitalisation and the frequency-weighted mean length of stay was 17.9 days. Mean DRG-level costs ranged from €5,859 to €22,495 per case, and mean length of stay ranged from 10.4 to 35.3 days (Table 1).

At pathway level, geriatric rehabilitation accounted for 20,318 hospitalisations, followed by septic hip/femur procedures (5,109), bone/joint infection (3,975), revision arthroplasty (3,954), soft-tissue reconstruction (2,467) and complex vacuum-assisted wound care (389) (Table 2).

**Table 2:** Frequency-weighted characteristics of the six predefined treatment pathways

Treatment pathway	DRGs	Cases	Weighted LOS (days)	Weighted cost/case (€)	Aggregate expenditure (€m)	Share (%)
Geriatric rehabilitation	I41Z	20,318	18.8	5,859	119.0	39.0
Septic hip/femur procedures	I08C, I08D	5,109	15.9	12,065	61.6	20.2
Revision arthroplasty	I01Z, I03B, I04Z	3,954	17.5	13,482	53.3	17.5
Bone/joint infection	I12A, I12B, I12C	3,975	13.2	8,065	32.1	10.5
Soft-tissue reconstruction	I22B, I50A	2,467	19.9	12,329	30.4	10.0
Complex VAC wound care	I98Z	389	35.3	22,495	8.8	2.9

Aggregate expenditure is the annual hospital-side expenditure represented by the included DRGs and must not be interpreted as the total national cost of implant-associated orthopaedic infection. LOS, length of stay; VAC, vacuum-assisted wound care

### Variation in hospital-side case costs

**Fig. 1:**
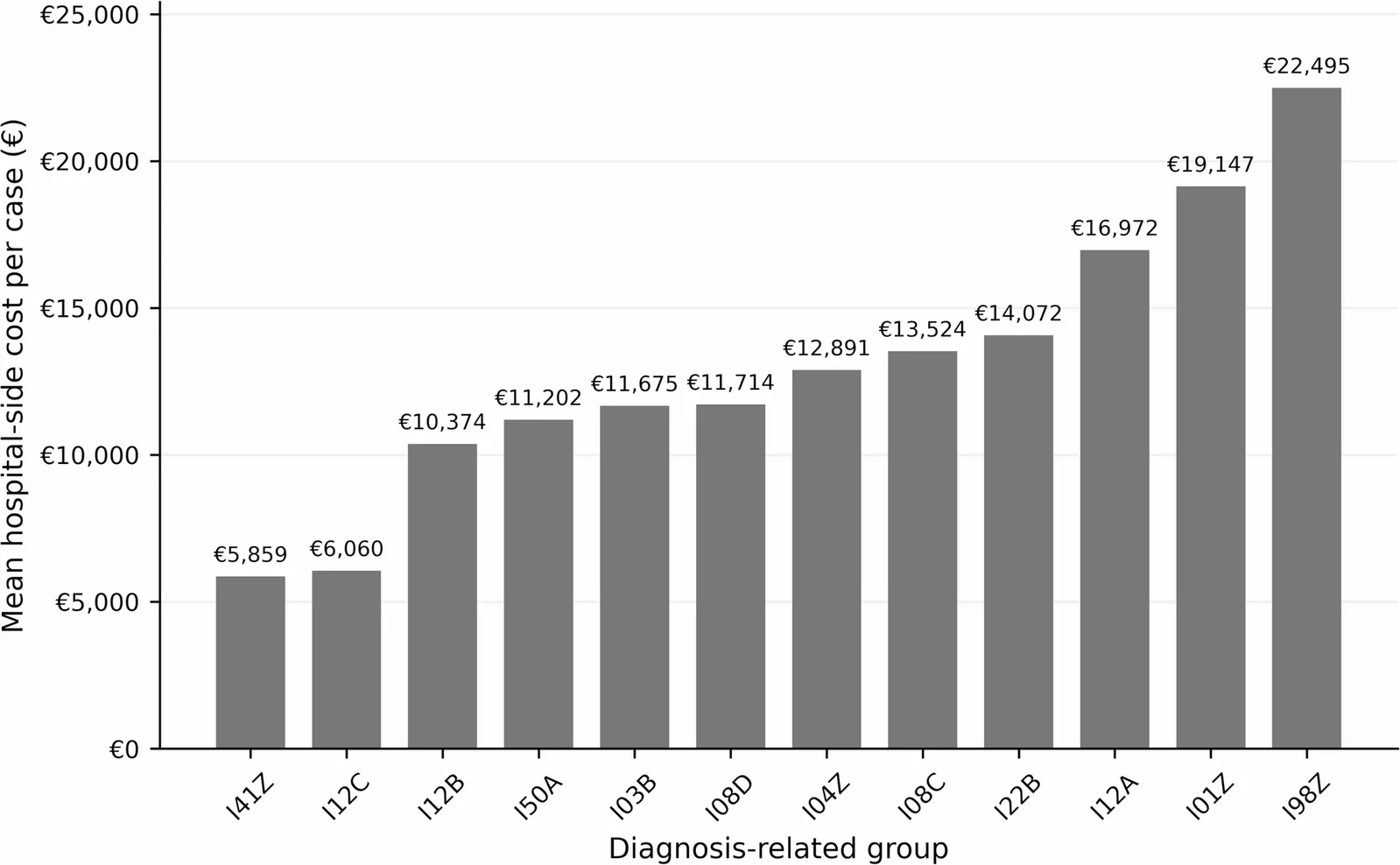
Mean hospital-side case costs across the twelve diagnosis-related groups (DRGs) included in the analysis. Costs represent standardised DRG-relevant hospital-side costs from the 2024 InEK cost matrix and do not represent actual DRG reimbursement

Hospital-side costs varied almost fourfold across the twelve DRGs (Fig. 1). Complex vacuum-assisted wound care (I98Z) had the highest mean cost (€22,495 per case), followed by I01Z (€19,147) and I12A (€16,972). Geriatric rehabilitation (I41Z) had the lowest mean cost (€5,859), while I12C was the lowest-cost infection-specific DRG (€6,060).

### Hospital cost-centre and cost-category composition

**Fig. 2:**
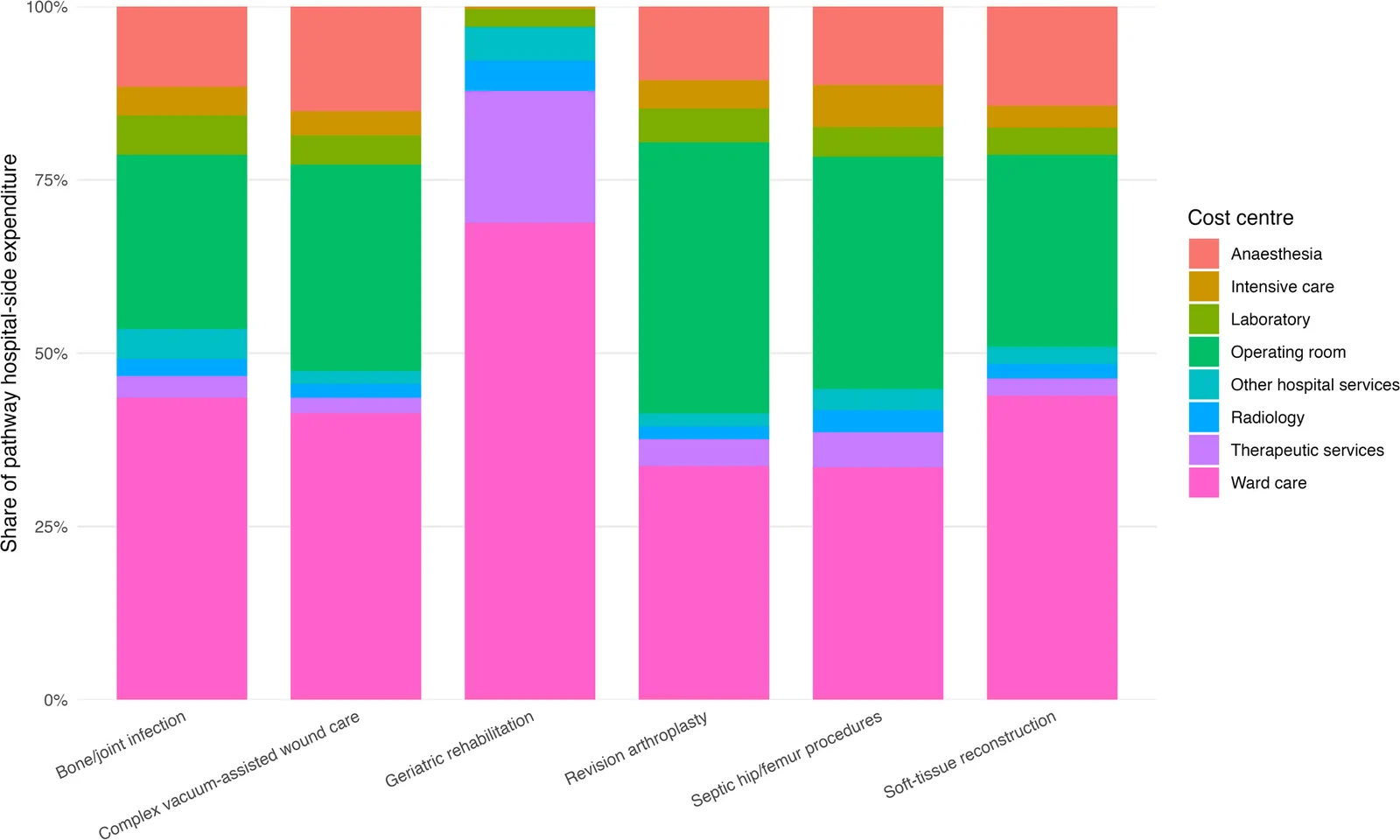
Hospital cost-centre composition across the six predefined treatment pathways. Pathway-level shares were calculated from absolute component expenditure. For each diagnosis-related group (DRG), mean component costs were multiplied by national hospitalisation frequencies, summed within each treatment pathway, and divided by the corresponding aggregate pathway hospital-side expenditure. Percentages therefore represent each cost centre’s share of total pathway-level hospital-side expenditure

Ward care was the largest cost centre across all six pathways, accounting for 33.5–68.8% of pathway expenditure (Fig. 2). Operating theatre expenditure was particularly prominent in revision arthroplasty (39.1%) and septic hip/femur procedures (33.5%), compared with 25.1% in bone/joint infection and 27.7% in soft-tissue reconstruction. Anaesthesia contributed approximately 10.6–15.1% across operative pathways, while intensive care accounted for approximately 3.1–6.1%.

Cost-category analysis reinforced these differences in resource profiles. Personnel and infrastructure constituted the largest components across pathways. Implant expenditure was most prominent in revision arthroplasty (13.6% of pathway expenditure), whereas it accounted for 8.5% in septic hip/femur procedures and approximately 2% or less in bone/joint infection, soft-tissue reconstruction, complex vacuum-assisted wound care and geriatric rehabilitation. Thus, high case costs were not explained by implant expenditure alone but reflected the combined use of ward, operative, anaesthetic, personnel and infrastructure resources.

### Per-case cost intensity versus aggregate expenditure

**Fig. 3:**
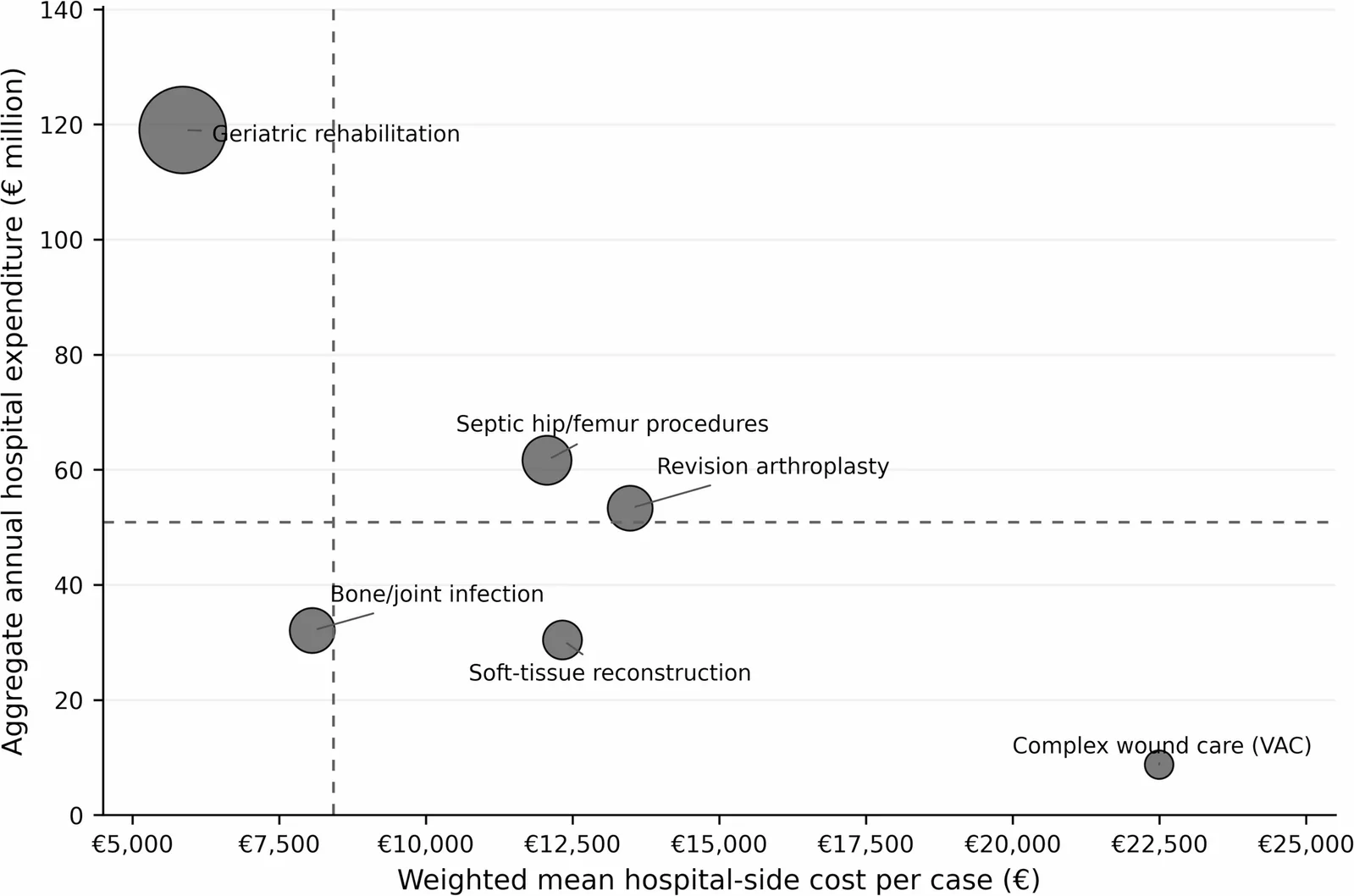
Relationship between weighted mean hospital-side cost per case and aggregate annual hospital-side expenditure represented by the included DRGs. Bubble size represents national hospitalisation volume. Aggregate expenditure was calculated as national hospitalisation frequency multiplied by mean hospital-side case cost. Dashed lines indicate the overall frequency-weighted mean case cost and the mean aggregate expenditure across the six pathways

Marked differences emerged between cost intensity per hospitalisation and aggregate expenditure represented by each pathway (Fig. 3). Geriatric rehabilitation generated the largest aggregate annual expenditure (€119.0 million; 39.0%) despite the lowest weighted mean cost per case (€5,859), reflecting its high national case volume. Septic hip/femur procedures were the second-largest contributor (€61.6 million; 20.2%), followed by revision arthroplasty (€53.3 million; 17.5%).

Conversely, complex vacuum-assisted wound care had the highest weighted mean cost per case (€22,495) but contributed only €8.8 million (2.9%) because of its low number of hospitalisations. Bone/joint infection and soft-tissue reconstruction accounted for €32.1 million (10.5%) and €30.4 million (10.0%), respectively. These estimates describe expenditure represented by the selected DRGs and do not constitute a disease-specific national burden estimate.

In a sensitivity analysis excluding geriatric rehabilitation (I41Z), the remaining five pathways represented 15,894 hospitalisations and €186.2 million in aggregate hospital-side expenditure. Septic hip/femur procedures accounted for the largest share (€61.6 million; 33.1%), followed by revision arthroplasty (€53.3 million; 28.6%), bone/joint infection (€32.1 million; 17.2%), soft-tissue reconstruction (€30.4 million; 16.3%) and complex vacuum-assisted wound care (€8.8 million; 4.7%) (Supplementary Table S2). Thus, exclusion of the high-volume, non-infection-specific geriatric rehabilitation DRG changed the relative expenditure distribution but did not alter the identification of septic hip/femur procedures and revision arthroplasty as the largest expenditure components among the remaining pathways.

### Association between length of stay and case costs

**Fig. 4:**
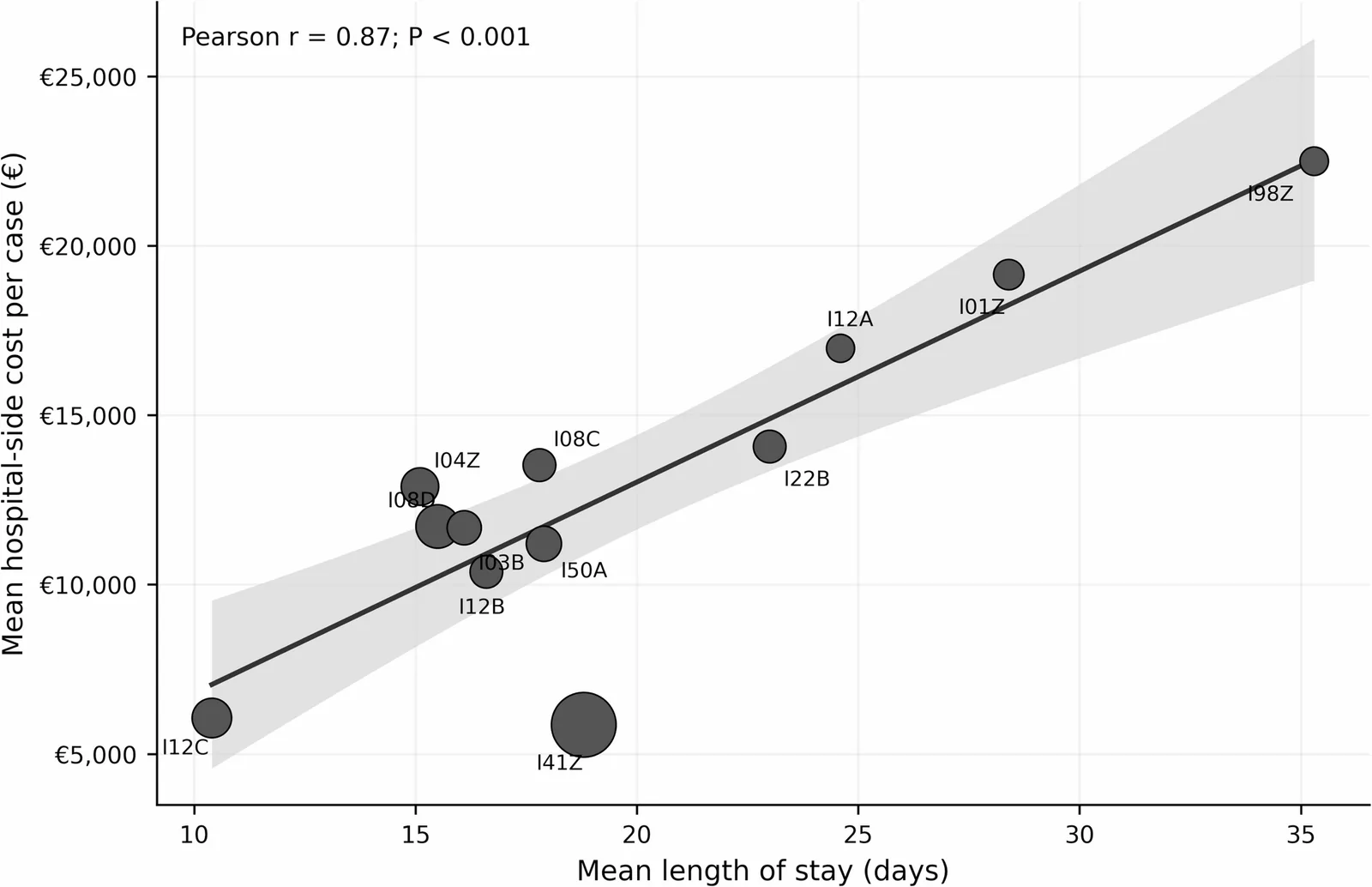
Association between mean length of stay and mean hospital-side case cost across the twelve included DRGs. Point size reflects national DRG case volume. The solid line represents the linear fit with a 95% confidence band. Pearson correlation was calculated using the twelve aggregated DRG-level observations; the analysis does not represent a patient-level association

Mean length of stay was strongly associated with mean hospital-side cost across the twelve DRGs (Pearson *r* = 0.87, 95% CI 0.60–0.96; *P* < 0.001; Fig. 4). The association remained statistically significant using Spearman’s rank correlation (ρ = 0.64, *P* = 0.024). In leave-one-out analyses, Pearson correlation coefficients ranged from *r* = 0.79 to *r* = 0.96, with all associations remaining statistically significant (all *P* < 0.005), indicating that the overall association was not driven by a single DRG. This DRG-level ecological association does not establish length of stay as an independent causal cost driver.

## Discussion

### Principal findings

This nationwide DRG-based analysis provides a detailed description of hospital cost structures across representative pathways relevant to complex orthopaedic infection care. Three findings are particularly important. First, mean hospital-side costs varied almost fourfold across the selected DRGs. Second, ward care dominated the cost structure across pathways, while operating theatre and anaesthesia made substantial contributions in surgically intensive groups. Third, longer mean hospital stay was strongly associated with higher mean case cost at DRG level.

### Economic burden and treatment-pathway complexity

The magnitude and heterogeneity of costs observed here are consistent with contemporary studies of PJI and FRI. The European PJI systematic review by Szymski et al. demonstrated substantial variation by treatment strategy, with two-stage exchange generally generating considerably greater costs than DAIR and, in many settings, one-stage exchange [5]. A more recent systematic review and meta-analysis of 24 studies estimated septic revision total hip arthroplasty to be 87% more costly than aseptic revision and two-stage septic revision to be 77% more costly than one-stage revision [12]. Patient-level work by Hammat et al. likewise found PJI to be the most expensive indication for revision total hip arthroplasty and reported median pathway costs of AU$123,688 for two-stage revision, compared with AU$63,261 for single-stage revision and AU$51,760 for debridement with implant retention [13]. Yao et al. showed that direct inpatient costs of two-stage hip and knee PJI treatment were approximately twice those of aseptic revision [14].

These treatment-strategy effects are clinically important but cannot be recovered reliably from aggregated DRG cost matrices. The same DRG may contain patients treated with implant retention, component removal or staged exchange, depending on diagnoses, procedures and case complexity. This limitation is central to interpretation of the present pathway-level estimates and explains why our analysis describes resource structures rather than comparative cost-effectiveness of surgical strategies. The present data therefore cannot be used to infer the relative clinical or economic value of DAIR, one-stage exchange, two-stage exchange or other specific infection-management strategies.

FRI studies show a similar pattern of high treatment intensity. Haidari et al. reported 3.1-fold higher hospital costs for FRI without recurrence and 7.6-fold higher costs for recurrent FRI, accompanied by five- to tenfold longer hospitalisation and additional infection-related operations [3]. Walter et al. reported 1.8- to 4.7-fold higher DRG-based costs for FRI treatment than for initial fracture care across several fracture locations in Germany [9]. Woffenden et al. found a 2.51-fold increase in healthcare costs, driven primarily by procedural and hospitalisation expenditure [15]. Iliaens et al. reported eightfold higher direct hospital-related costs and substantial indirect costs associated with absenteeism [16]. These findings are also aligned with the 2026 systematic review by Stolz et al., which found a median relative cost increase of 120% for orthopaedic and trauma surgical-site infections after adjustment for other cost-influencing factors [4].

### Ward care, operative resources and length of stay

The cost-centre composition in our analysis helps explain why infection-related treatment remains expensive even when implant expenditure is modest. Nyffeler et al. found that hospitalisation-related expenditure, including nursing and physiotherapy, accounted for 49% of FRI treatment costs, compared with 19% for surgical procedures; duration of intravenous antimicrobial therapy and changes in surgical strategy were associated with higher expenditure [17]. In a multicentre cost-of-illness study of open lower-limb fractures, Noorlander-Borgdorff et al. attributed 58% of one-year hospital costs to length of stay and 30% to surgery, with total costs markedly higher in infected cases [18].

Our finding that ward care accounted for approximately one-third to more than two-thirds of pathway expenditure is therefore clinically plausible. At the same time, the high operating-theatre share in revision arthroplasty and septic hip/femur procedures underscores the dual resource burden of prolonged inpatient care and repeated complex surgery. PJI studies provide similar evidence. Kapadia et al. reported substantially longer hospitalisation, more readmissions and approximately fourfold higher annual costs after infected total knee arthroplasty than after uncomplicated primary arthroplasty [19], while Morcos et al. found an approximately fivefold cost increase for two-stage revision total knee arthroplasty [20]. German hospital-level data likewise demonstrate markedly greater length of stay and direct costs for two-stage septic compared with aseptic knee revision [21].

The strong correlation between mean length of stay and mean case costs in our dataset should not be interpreted causally. DRG-level averages combine differences in case mix, comorbidity, procedure intensity and discharge pathways. The leave-one-out analysis showed that the association remained present after sequential exclusion of each individual DRG, although this sensitivity analysis does not overcome the ecological nature or small number of aggregated observations. Nevertheless, the consistency of our ecological association with patient-level studies supports inpatient duration as an important marker of resource intensity. Importantly, I41Z illustrates that length of stay alone is insufficient: despite an intermediate mean stay, its per-case cost was low because the pathway was dominated by ward and therapeutic services with minimal operative activity.

### Cost intensity, treatment volume and implications for hospital planning

For hospital resource planning, per-case cost intensity should be considered together with treatment volume when interpreting aggregate expenditure. High-cost cases may contribute relatively little to overall expenditure when their frequency is low. Premkumar et al. projected that increasing PJI treatment volume, rather than major increases in per-case expenditure, would be a key determinant of future US hospital costs [7]. At European level, Alt et al. similarly showed that the aggregate reimbursement burden of PJI reflects both treatment frequency and the distribution of DAIR, one-stage and two-stage procedures [8].

In our selected DRGs, complex vacuum-assisted wound care represented the greatest per-case resource intensity but only a small share of aggregate expenditure. In contrast, the high-volume geriatric rehabilitation DRG generated the largest aggregate expenditure despite the lowest cost per case. Because I41Z is not infection-specific, we repeated the expenditure analysis after excluding this DRG. Among the remaining pathways, septic hip/femur procedures and revision arthroplasty accounted for the largest shares of aggregate hospital-side expenditure (33.1% and 28.6%, respectively). This does not imply that geriatric rehabilitation expenditure is attributable specifically to implant-associated infection; rather, it demonstrates why pathway volume must be considered alongside cost intensity when planning bed capacity, rehabilitation services, operating resources and specialist staffing.

### Implications for the German DRG system

The distinction between cost and reimbursement is essential. The InEK cost matrix describes standardised DRG-relevant costs reported by calculation hospitals, whereas actual DRG remuneration depends on cost weights and base rates [1, 2]. Consequently, the present analysis does not demonstrate under- or over-reimbursement. It instead identifies where hospital resources are concentrated and provides a cost-structure basis for subsequent cost–revenue analyses.

This is particularly relevant in trauma and orthopaedic surgery, where resource intensity may be driven by operating-theatre time, implants, prolonged ward treatment, intensive care and multidisciplinary rehabilitation in different proportions. Recent EJTES work comparing acute fracture procedures with elective arthroplasty demonstrated that the relationship between DRG remuneration, length of stay, operating time, implant costs and contribution margins can vary substantially across procedures [22]. Similarly, studies from other European reimbursement systems have shown that the relationship between PJI treatment costs and reimbursement is highly dependent on strategy and institutional context [23]. A direct assessment of reimbursement adequacy in septic orthopaedic care therefore requires patient-level linkage of actual hospital costs, procedure sequences and realised revenues rather than extrapolation from the InEK cost matrix alone.

### Strengths, limitations and future research

The principal strength of this study is the use of a uniform national cost-accounting framework that permits direct comparison of hospital resource structures across multiple clinically relevant DRGs. The analysis combines case volume, mean hospital-side costs, length of stay, cost-centre composition, cost categories and aggregate expenditure within one framework. The inclusion of recent national frequencies and standardised InEK costing also reduces the institutional heterogeneity inherent in single-centre cost studies.

Several limitations require emphasis. First, the data are aggregated at DRG level and contain no patient-level clinical outcomes, longitudinal episodes, comorbidity adjustment or microbiological information. The InEK data provide DRG-level mean costs and mean length of stay but no patient-level distributions; therefore, patient-level medians and interquartile ranges could not be calculated. In particular, recurrence, readmission, mortality and health-related quality of life could not be evaluated. Most importantly, the aggregate DRG structure does not permit reliable differentiation between implant retention/DAIR, implant removal, one-stage exchange and two-stage exchange. Surgical strategies may coexist within a DRG because grouping is determined by the combination of diagnoses, procedures and complexity rather than by a single treatment strategy. This precludes direct strategy-specific cost comparisons.

Second, although several DRGs are anatomically specific—for example, hip- and knee-related revision groups—consistent stratification by anatomical site was not possible across all pathways. Separate hip-versus-knee estimates would therefore have produced an incomplete and potentially misleading comparison. Third, the selected DRGs were chosen to represent resource-intensive components of orthopaedic infection care but are not infection-exclusive. Accordingly, the €305.2 million aggregate expenditure represented by these groups must not be interpreted as the total national economic burden of implant-associated orthopaedic infection. This limitation is particularly relevant to I41Z, which represents geriatric early rehabilitation for musculoskeletal disorders without an infection-specific requirement; its influence was therefore examined in a separate sensitivity analysis. Fourth, InEK cost data are derived from participating calculation hospitals and represent standardised hospital-side cost structures rather than the realised costs of every German hospital. Furthermore, the findings are specific to the German G-DRG and InEK cost-accounting framework and may not be directly generalisable to healthcare systems with different reimbursement mechanisms, costing methods or patterns of inpatient care. Outpatient expenditure, post-discharge rehabilitation outside the represented DRG episode, societal costs and productivity losses were not captured. Finally, the cross-sectional ecological design does not allow causal inference regarding individual cost drivers. The correlation analysis was based on only twelve aggregated DRGs and should therefore be regarded as exploratory rather than as evidence of a patient-level relationship between length of stay and costs.

Future studies should use patient-level statutory health-insurance claims or linked hospital datasets combining ICD-10-GM diagnoses with OPS procedure codes and longitudinal admissions. Such data would allow explicit identification of DAIR, one-stage and two-stage pathways, separate hip and knee analyses, adjustment for comorbidity and infection severity, and comparison of cumulative treatment costs with actual reimbursement. This would provide the strategy-level resolution that aggregated DRG cost matrices cannot offer.

## Conclusion

Hospital resource utilisation differs substantially across representative DRG pathways relevant to complex orthopaedic infection management in Germany. Ward care was the dominant cost centre across pathways, while operating-theatre and perioperative resources made major contributions to surgically intensive groups. The aggregate expenditure estimates describe hospital-side expenditure represented by the selected DRGs; because these DRGs are not infection-exclusive, they should not be interpreted as the national economic burden of implant-associated orthopaedic infection. The InEK cost matrix provides a valuable framework for describing these resource structures, but it should not be interpreted as actual reimbursement. Associations between length of stay and costs were observed only at the aggregated DRG level and should not be interpreted causally at patient level. Patient-level linkage of diagnoses, procedures, clinical outcomes, costs and revenues is required to evaluate strategy-specific costs, comparative value and reimbursement adequacy in septic orthopaedic care.

## Supplementary Information

Below is the link to the electronic supplementary material.

Supplementary Material 1

Supplementary Material 2

Supplementary Material 3

Supplementary Material 4

## Data Availability

The data used in this study are publicly available, aggregated administrative data obtained from the German Institute for the Hospital Remuneration System (InEK). No individual-level patient data were used.
