# Supplementary material for: Hospital cost structures and resource utilisation across representative treatment pathways relevant to complex orthopaedic infection care: a nationwide DRG-based analysis in Germany
Source: Eur J Trauma Emerg Surg. 2026 Nov 10;52(1):339. doi: 10.1007/s00068-026-03391-2 (PMC13751573; doi:10.1007/s00068-026-03391-2)
Supplement: Supplementary file 1 [file 68_2026_3391_MOESM1_ESM.docx]

**Hospital Cost Structures and Resource Utilisation Across Representative Treatment Pathways Relevant to Complex Orthopaedic Infection Care: A Nationwide DRG-Based Analysis in Germany**

Houmam Anees^1,2^*****, Goran Georgievski^1^, Jonas Pawelke^1,2^, Matthias Muelke^1^, Gero Knapp^1^, Thaqif El Khassawna^2,3^, Markus Rupp^1,2^, and Christian Heiss^1,2,4^

^1^Department of Trauma, Hand and Reconstructive Surgery, Faculty of Medicine, Justus-Liebig-University of Giessen, 35392, Giessen, Germany

^2^Experimental Trauma Surgery, Faculty of Medicine, Justus-Liebig-University of Giessen, 35392, Giessen, Germany

^3^School of Pharmacy, The University of Jordan, Amman 11942, Jordan

^4^Biruni University, Istanbul, Türkiye

***Corresponding Author:** [Houmam.Anees@chiru.med.uni-giessen.de](mailto:Houmam.Anees@chiru.med.uni-giessen.de) (H. Anees).

**Supplementary Table S1.** Clinical rationale and InEK evidence for selection of the twelve representative DRGs

All diagnostic and procedural percentages were verified directly against the 2024 InEK Data Browser exports. Percentages correspond to the InEK “Prozent (F)” field, i.e. the proportion of cases within the respective DRG in which the diagnosis or procedure was reported. Principal and secondary diagnoses are identified explicitly.

| **DRG** | **Treatment pathway** | **Official 2024 G-DRG definition (German)** | **Verified diagnostic/procedural evidence from InEK Data Browser** | **Reason for inclusion / caveat** |
| --- | --- | --- | --- | --- |
| **I01Z** | Revision arthroplasty | Beidseitige Eingriffe oder mehrere große Eingriffe an Gelenken der unteren Extremität mit komplexer Diagnose | Secondary diagnosis T84.5 in 70.8% of cases; OPS 5-800.3g hip-joint debridement in 35.7%; OPS 5-800.2g septic hip-joint lavage with drainage in 16.6%. | Complex lower-extremity joint procedures with a strong PJI-related diagnostic and procedural signal; not infection-exclusive. |
| **I03B** | Revision arthroplasty | Revision oder Ersatz des Hüftgelenkes mit kompl. Diagnose od. Arthrodese od. Alter < 16 Jahre oder beidseitige od. mehrere gr. Eingr. an Gelenken der unt. Extr. mit kompl. Eingriff, ohne äuß. schw. CC, ohne mehrzeit. Wechsel, ohne Eingr. an mehr. Lok. | Secondary diagnosis T84.5 in 35.8% of cases; OPS 5-800.3g hip-joint debridement in 23.1%; OPS 5-800.2g septic hip-joint lavage with drainage in 9.8%; OPS 5-821.7 removal of a total hip endoprosthesis in 9.8%. | Complex hip revision/replacement pathway with substantial PJI-related diagnostic and procedural content; not infection-exclusive. |
| **I04Z** | Revision arthroplasty | Implantation, Wechsel oder Entfernung einer Endoprothese am Kniegelenk mit komplizierender Diagnose oder Arthrodese oder Implantation einer Endoprothese nach vorheriger Explantation oder periprothetische Fraktur an der Schulter oder am Knie | Secondary diagnosis T84.5 in 47.4% of cases; OPS 5-829.n endoprosthesis implantation after previous explantation in 51.7%; OPS 5-829.g spacer removal in 40.8%; OPS 5-800.3h knee-joint debridement in 21.5%. | Complex knee revision/exchange/removal pathway including staged reconstruction features; not infection-exclusive. |
| **I08C** | Septic hip/femur procedures | Andere Eingriffe an Hüftgelenk und Femur mit Einbringen von Abstandshaltern od. and. komplexen Eingriffen od. äuß. schw. CC od. bei kompl. Diagnose od. Ersatz des Hüftgelenks mit Eingriff an oberer Extremität od. Wirbelsäule ohne best. kompliz. Faktoren | Secondary diagnosis T84.5 in 25.6% of cases; OPS 5-829.9 spacer insertion in 36.5%; OPS 5-821.7 removal of a total hip endoprosthesis in 18.3%; OPS 5-800.3g hip-joint debridement in 16.0%. | Complex hip/femur procedures with spacer, explantation and debridement features relevant to septic reconstruction; not infection-exclusive. |
| **I08D** | Septic hip/femur procedures | Andere Eingriffe an Hüftgelenk und Femur mit komplexer Diagnose oder Prozedur oder äußerst schweren CC | Principal diagnosis T84.5 in 3.6% of cases; secondary diagnosis T84.5 in 7.4%; OPS 5-800.3g hip-joint debridement in 10.3%. | Broader complex hip/femur pathway with an identifiable infection-related subgroup; lower infection specificity than I08C and therefore interpreted cautiously. |
| **I12A** | Bone/joint infection | Knochen- und Gelenkinfektion / -entzündung mit verschiedenen Eingriffen am Muskel-Skelett-System und Bindegewebe mit äußerst schweren CC | Official DRG definition explicitly specifies bone/joint infection. Secondary diagnosis T84.5 in 38.6% of cases and T84.6 in 12.7%; OPS 5-829.9 spacer insertion in 15.6%; OPS 5-810.1h arthroscopic septic knee-joint lavage with drainage in 13.0%. | Explicit infection DRG with very high complexity and procedures directly relevant to implant-associated infection. |
| **I12B** | Bone/joint infection | Knochen- und Gelenkinfektion / -entzündung mit verschiedenen Eingriffen am Muskel-Skelett-System und Bindegewebe mit schweren CC, mit Revision des Kniegelenkes, mit Einbringen oder Wechsel von Abstandshaltern oder Osteomyelitis, Alter < 16 Jahre | Official DRG definition explicitly specifies bone/joint infection and revision/spacer/osteomyelitis features. Secondary diagnosis T84.5 in 29.3% of cases; OPS 5-800.3h knee-joint debridement in 19.0%; OPS 5-800.2h open septic knee-joint lavage with drainage in 18.3%; OPS 5-829.9 spacer insertion in 17.2%. | Explicit infection DRG incorporating revision, spacer and osteomyelitis features. |
| **I12C** | Bone/joint infection | Knochen- und Gelenkinfektion / -entzündung mit verschiedenen Eingriffen am Muskel-Skelett-System und Bindegewebe mit schweren CC, ohne Revision des Kniegelenkes, ohne Einbringen oder Wechsel von Abstandshaltern, ohne Osteomyelitis, Alter > 15 Jahre | Official DRG definition explicitly specifies bone/joint infection. Principal diagnosis T84.6 in 24.5% of cases; OPS 5-810.1h arthroscopic septic knee-joint lavage with drainage in 28.8%; OPS 5-787.3r plate removal at the distal fibula in 4.2% and OPS 5-780.6r bone debridement at the distal fibula in 4.1%. | Explicit infection DRG without the revision/spacer/osteomyelitis features defining I12B; represents a lower-complexity infection pathway. |
| **I22B** | Soft-tissue reconstruction | Gewebe- / Hauttransplantation, außer an der Hand, mit kleinflächiger Gewebetransplantation od. mit großflächiger Gewebetransplantation ohne kompliz. Konst., oh. Eingr. an mehreren Lokal., oh. schw. Weichteilschaden, oh. kompl. Gewebetranspl. m. schw. CC | Principal diagnosis T84.6 in 5.3% of cases; OPS 5-916.a1 deep VAC-system application/change in 54.9%; OPS 5-916.a0 VAC-system application/change at skin/subcutaneous tissue in 42.2%; OPS 8-190.21 continuous suction VAC for 8–14 days in 33.1%. | Represents soft-tissue reconstruction and VAC treatment commonly required in complex infection care; not infection-exclusive. |
| **I50A** | Soft-tissue reconstruction | Gewebe- / Haut-Transplantation außer an der Hand, ohne bestimmte komplizierende Faktoren, mit bestimmtem Eingriff oder bestimmter Vakuumbehandlung mit kontinuierlicher Sogbehandlung ab 8 Tagen | Principal diagnosis T84.6 in 11.0% of cases; OPS 8-190.21 continuous suction VAC for 8–14 days in 54.5%; OPS 5-916.a1 deep VAC-system application/change in 54.3%; OPS 5-896.1f extensive wound debridement of the lower leg in 21.6%. | Represents prolonged VAC and reconstructive wound treatment relevant to FRI/implant-associated infection care; not infection-exclusive. |
| **I98Z** | Complex VAC wound care | Komplexe Vakuumbehandlung bei Krankheiten und Störungen an Muskel-Skelett-System und Bindegewebe | Principal diagnosis T84.6 in 6.9% of cases and M86.48 chronic osteomyelitis with fistula in 4.4%; OPS 5-916.a1 deep VAC-system application/change in 73.0%; OPS 8-190.22 continuous suction VAC for 15–21 days in 59.4%. | Represents highly resource-intensive prolonged VAC treatment relevant to complex septic wound care; not infection-exclusive. |
| **I41Z** | Geriatric rehabilitation | Geriatrische frührehabilitative Komplexbehandlung bei Krankheiten und Störungen an Muskel-Skelett-System und Bindegewebe | OPS 8-550.1 geriatric early rehabilitation (at least 14 treatment days and 20 therapy units) in 96.1% of cases; the DRG definition itself contains no infection-specific requirement. | Included as a downstream/high-volume rehabilitation pathway relevant to complex musculoskeletal care, but not infection-specific; therefore additionally examined by sensitivity analysis excluding I41Z. |

**Abbreviations:** CC, complications or comorbidities; DRG, diagnosis-related group; InEK, Institut für das Entgeltsystem im Krankenhaus; OPS, Operationen- und Prozedurenschlüssel; PJI, periprosthetic joint infection; FRI, fracture-related infection; VAC, vacuum-assisted closure/wound therapy.
