# Supplementary material for: Hospital cost structures and resource utilisation across representative treatment pathways relevant to complex orthopaedic infection care: a nationwide DRG-based analysis in Germany
Source: Eur J Trauma Emerg Surg. 2026 Nov 10;52(1):339. doi: 10.1007/s00068-026-03391-2 (PMC13751573; doi:10.1007/s00068-026-03391-2)
Supplement: Supplementary file 2 [file 68_2026_3391_MOESM2_ESM.docx]

**Hospital Cost Structures and Resource Utilisation Across Representative Treatment Pathways Relevant to Complex Orthopaedic Infection Care: A Nationwide DRG-Based Analysis in Germany**

Houmam Anees^1,2^*****, Goran Georgievski^1^, Jonas Pawelke^1,2^, Matthias Muelke^1^, Gero Knapp^1^, Thaqif El Khassawna^2,3^, Markus Rupp^1,2^, and Christian Heiss^1,2,4^

^1^Department of Trauma, Hand and Reconstructive Surgery, Faculty of Medicine, Justus-Liebig-University of Giessen, 35392, Giessen, Germany

^2^Experimental Trauma Surgery, Faculty of Medicine, Justus-Liebig-University of Giessen, 35392, Giessen, Germany

^3^School of Pharmacy, The University of Jordan, Amman 11942, Jordan

^4^Biruni University, Istanbul, Türkiye

***Corresponding Author:** [Houmam.Anees@chiru.med.uni-giessen.de](mailto:Houmam.Anees@chiru.med.uni-giessen.de) (H. Anees).

**Supplementary Table S2. Sensitivity analysis of aggregate hospital-side expenditure after exclusion of geriatric rehabilitation (I41Z).** Aggregate expenditure was calculated by multiplying national hospitalisation frequencies by the corresponding mean hospital-side case cost for each diagnosis-related group (DRG) and summing across DRGs within each treatment pathway. Expenditure shares were recalculated relative to the total expenditure of the five remaining pathways after exclusion of I41Z. Values are rounded for presentation; therefore, displayed pathway percentages may not sum exactly to 100%. DRG, diagnosis-related group; VAC, vacuum-assisted wound care.

| **Treatment pathway** | **DRGs** | **Cases** | **Aggregate hospital-side expenditure (€m)** | **Share after exclusion of I41Z (%)** |
| --- | --- | --- | --- | --- |
| Septic hip/femur procedures | I08C, I08D | 5,109 | 61.6 | 33.1 |
| Revision arthroplasty | I01Z, I03B, I04Z | 3,954 | 53.3 | 28.6 |
| Bone/joint infection | I12A, I12B, I12C | 3,975 | 32.1 | 17.2 |
| Soft-tissue reconstruction | I22B, I50A | 2,467 | 30.4 | 16.3 |
| Complex VAC wound care | I98Z | 389 | 8.8 | 4.7 |
| **Total** |  | **15,894** | **186.2** | **100.0** |
