# Supplementary material for: Hospital cost structures and resource utilisation across representative treatment pathways relevant to complex orthopaedic infection care: a nationwide DRG-based analysis in Germany
Source: Eur J Trauma Emerg Surg. 2026 Nov 10;52(1):339. doi: 10.1007/s00068-026-03391-2 (PMC13751573; doi:10.1007/s00068-026-03391-2)
Supplement: Supplementary file 3 [file 68_2026_3391_MOESM3_ESM.docx]

**Hospital Cost Structures and Resource Utilisation Across Representative Treatment Pathways Relevant to Complex Orthopaedic Infection Care: A Nationwide DRG-Based Analysis in Germany**

Houmam Anees^1,2^*****, Goran Georgievski^1^, Jonas Pawelke^1,2^, Matthias Muelke^1^, Gero Knapp^1^, Thaqif El Khassawna^2,3^, Markus Rupp^1,2^, and Christian Heiss^1,2,4^

^1^Department of Trauma, Hand and Reconstructive Surgery, Faculty of Medicine, Justus-Liebig-University of Giessen, 35392, Giessen, Germany

^2^Experimental Trauma Surgery, Faculty of Medicine, Justus-Liebig-University of Giessen, 35392, Giessen, Germany

^3^School of Pharmacy, The University of Jordan, Amman 11942, Jordan

^4^Biruni University, Istanbul, Türkiye

***Corresponding Author:** [Houmam.Anees@chiru.med.uni-giessen.de](mailto:Houmam.Anees@chiru.med.uni-giessen.de) (H. Anees).

**Supplementary Table S3.** DRG-level hospital cost-centre components. Values are mean hospital-side case costs (€ per case) taken directly from the 2024 InEK cost matrix. Cost-centre values correspond to the InEK cost-centre 'Summe' values for each DRG. The total column reproduces the DRG-level mean hospital-side case cost. Values are shown to two decimal places and may therefore differ from rounded values reported in the main manuscript. DRG, diagnosis-related group; ICU, intensive care unit; InEK, Institut für das Entgeltsystem im Krankenhaus; OR, operating theatre.

| **DRG** | **Ward care (01)** | **ICU (02)** | **Operating theatre (04)** | **Anaesthesia (05)** | **Cardiology diag./therapy (07)** | **Endoscopy diag./therapy (08)** | **Radiology (09)** | **Laboratories (10)** | **Diagnostic areas (11)** | **Therapeutic procedures (12)** | **Patient admission (13)** | **Total (€)** |
| --- | --- | --- | --- | --- | --- | --- | --- | --- | --- | --- | --- | --- |
| **I01Z** | 7,168.40 | 893.87 | 6,411.11 | 2,094.97 | 1.11 | 18.27 | 378.84 | 984.67 | 100.17 | 811.92 | 283.72 | **19,147.05** |
| **I03B** | 4,162.91 | 426.80 | 4,226.74 | 1,307.10 | 3.06 | 5.42 | 270.85 | 577.56 | 45.47 | 472.40 | 176.25 | **11,674.56** |
| **I04Z** | 3,997.07 | 526.13 | 5,569.56 | 1,312.24 | 4.84 | 5.54 | 200.19 | 610.01 | 54.50 | 458.42 | 152.56 | **12,891.06** |
| **I08C** | 4,562.49 | 804.21 | 4,575.13 | 1,563.88 | 28.82 | 21.50 | 392.13 | 632.92 | 74.79 | 630.07 | 237.86 | **13,523.80** |
| **I08D** | 3,921.76 | 718.23 | 3,914.83 | 1,314.64 | 19.89 | 23.83 | 386.36 | 487.59 | 74.41 | 599.86 | 252.78 | **11,714.18** |
| **I12A** | 6,230.05 | 1,953.94 | 3,981.37 | 1,626.05 | 35.06 | 92.22 | 520.77 | 1,361.27 | 190.41 | 637.88 | 342.69 | **16,971.71** |
| **I12B** | 4,473.53 | 487.40 | 2,690.03 | 1,099.87 | 19.51 | 20.58 | 267.82 | 589.54 | 87.78 | 350.64 | 287.53 | **10,374.23** |
| **I12C** | 2,810.13 | 65.69 | 1,526.62 | 782.24 | 0.78 | 6.36 | 134.53 | 292.76 | 37.22 | 166.62 | 237.15 | **6,060.10** |
| **I22B** | 6,121.27 | 682.15 | 3,576.06 | 1,841.20 | 24.37 | 24.40 | 365.55 | 670.08 | 100.57 | 417.91 | 248.21 | **14,071.77** |
| **I41Z** | 4,030.34 | 20.04 | 4.77 | 2.69 | 4.41 | 35.55 | 257.34 | 148.56 | 119.55 | 1,113.20 | 122.17 | **5,858.62** |
| **I50A** | 4,947.21 | 195.85 | 3,303.26 | 1,720.91 | 1.36 | 5.05 | 187.50 | 364.47 | 47.41 | 229.09 | 199.66 | **11,201.77** |
| **I98Z** | 9,302.38 | 782.16 | 6,688.23 | 3,397.05 | 13.52 | 25.84 | 456.40 | 952.64 | 111.98 | 492.64 | 271.71 | **22,494.55** |
