# Supplementary material for: Hospital cost structures and resource utilisation across representative treatment pathways relevant to complex orthopaedic infection care: a nationwide DRG-based analysis in Germany
Source: Eur J Trauma Emerg Surg. 2026 Nov 10;52(1):339. doi: 10.1007/s00068-026-03391-2 (PMC13751573; doi:10.1007/s00068-026-03391-2)
Supplement: Supplementary file 4 [file 68_2026_3391_MOESM4_ESM.docx]

**Hospital Cost Structures and Resource Utilisation Across Representative Treatment Pathways Relevant to Complex Orthopaedic Infection Care: A Nationwide DRG-Based Analysis in Germany**

Houmam Anees^1,2^*****, Goran Georgievski^1^, Jonas Pawelke^1,2^, Matthias Muelke^1^, Gero Knapp^1^, Thaqif El Khassawna^2,3^, Markus Rupp^1,2^, and Christian Heiss^1,2,4^

^1^Department of Trauma, Hand and Reconstructive Surgery, Faculty of Medicine, Justus-Liebig-University of Giessen, 35392, Giessen, Germany

^2^Experimental Trauma Surgery, Faculty of Medicine, Justus-Liebig-University of Giessen, 35392, Giessen, Germany

^3^School of Pharmacy, The University of Jordan, Amman 11942, Jordan

^4^Biruni University, Istanbul, Türkiye

***Corresponding Author:** [Houmam.Anees@chiru.med.uni-giessen.de](mailto:Houmam.Anees@chiru.med.uni-giessen.de) (H. Anees).

**Supplementary Table S4.** DRG-level hospital cost-category components. Values are mean hospital-side case costs (€ per case) taken directly from the 2024 InEK cost matrix. Cost categories correspond to InEK groups 1, 2, 3, 4a, 4b, 5, 6a, 6b, 6c, 7 and 8; the component sum equals the total mean hospital-side case cost for each DRG. Values are shown to two decimal places and may therefore differ from rounded values reported in the main manuscript. DRG, diagnosis-related group; InEK, Institut für das Entgeltsystem im Krankenhaus.

| **DRG** | **Physician personnel (1)** | **Nursing personnel (2)** | **Medical technical / functional service (3)** | **Pharmaceuticals, overhead (4a)** | **Pharmaceuticals, case-specific (4b)** | **Implants / transplants (5)** | **Other medical supplies, overhead (6a)** | **Other medical supplies, case-specific (6b)** | **Externally purchased medical services (6c)** | **Medical infrastructure (7)** | **Non-medical infrastructure (8)** | **Total (€)** |
| --- | --- | --- | --- | --- | --- | --- | --- | --- | --- | --- | --- | --- |
| I01Z | 4,969.41 | 56.32 | 2,896.55 | 357.87 | 349.22 | 1,791.80 | 1,204.11 | 202.71 | 396.74 | 2,093.00 | 4,829.32 | **19,147.05** |
| I03B | 2,970.33 | 33.40 | 1,677.62 | 233.75 | 192.67 | 1,165.75 | 687.94 | 132.54 | 375.56 | 1,277.93 | 2,927.07 | **11,674.56** |
| I04Z | 2,931.04 | 27.95 | 1,755.90 | 230.57 | 260.43 | 2,264.42 | 727.08 | 187.09 | 257.88 | 1,309.18 | 2,939.52 | **12,891.06** |
| I08C | 3,621.44 | 43.32 | 2,193.72 | 270.05 | 262.86 | 954.97 | 838.86 | 172.94 | 273.59 | 1,504.71 | 3,387.34 | **13,523.80** |
| I08D | 3,076.69 | 40.95 | 1,896.89 | 223.15 | 191.21 | 1,043.65 | 729.70 | 148.76 | 208.13 | 1,275.62 | 2,879.43 | **11,714.18** |
| I12A | 4,575.11 | 46.09 | 2,509.36 | 422.36 | 585.58 | 652.03 | 1,196.15 | 195.27 | 455.45 | 1,951.66 | 4,382.65 | **16,971.71** |
| I12B | 2,867.58 | 52.83 | 1,573.43 | 217.05 | 122.00 | 340.77 | 673.82 | 136.78 | 262.14 | 1,225.87 | 2,901.96 | **10,374.23** |
| I12C | 1,727.72 | 31.14 | 979.08 | 122.25 | 25.77 | 31.66 | 388.10 | 78.51 | 143.75 | 757.48 | 1,774.64 | **6,060.10** |
| I22B | 4,165.49 | 43.78 | 2,206.24 | 306.33 | 212.96 | 266.67 | 946.83 | 203.99 | 260.38 | 1,613.20 | 3,845.90 | **14,071.77** |
| I41Z | 1,453.00 | 58.09 | 1,103.51 | 125.86 | 16.03 | 0.29 | 180.80 | 2.97 | 199.17 | 563.05 | 2,155.85 | **5,858.62** |
| I50A | 3,479.24 | 32.67 | 1,827.72 | 212.18 | 48.44 | 136.59 | 753.53 | 174.46 | 158.04 | 1,344.72 | 3,034.18 | **11,201.77** |
| I98Z | 6,888.85 | 50.82 | 3,608.87 | 501.33 | 185.98 | 421.69 | 1,628.52 | 423.58 | 384.02 | 2,525.40 | 5,875.49 | **22,494.55** |
